# FGF13 prevents age-related hearing loss by protecting spiral ganglion neurons and ribbon synapses from injury

**DOI:** 10.1038/s41420-025-02607-5

**Published:** 2025-07-05

**Authors:** Huan Yin, Huan Cao, Jianwang Yang, Tao Liu, Qi Li, Mengxiao Liu, Baoshan Wang

**Affiliations:** Department of Otolaryngology-Head and Neck Surgery, The Second Hospital of Hebei Medical University, Shijiazhuang, China

**Keywords:** Neurodegeneration, Cochlea

## Abstract

Age-related hearing loss (ARHL) is the most common sensorineural hearing loss, and the dysfunction of spiral ganglion neurons (SGNs) and ribbon synapses plays a crucial role in the pathogenesis. The fibroblast growth factor 13 (FGF13) is considered to be associated with neuronal survival and synaptic transmission. However, whether FGF13 is involved in degeneration of SGNs and ribbon synapses, the typical changes of ARHL, is still unknown. Firstly, the expression of FGF13 mRNA and protein, was all dramatically decreased in the SGNs of aged mice, accompanied by impaired SGNs and ribbon synapses. More importantly, specific upregulation of FGF13 in SGNs significantly reduced hearing threshold, improved wave I amplitude, and alleviated loss of SGNs as well as ribbon synapses. Furthermore, the proteomic analysis and verification results suggested that the decrease of FGF13 induced the loss of SGNs and ribbon synapses partly by regulating the ORC1. Taken together, our data revealed that FGF13 might protect SGNs and ribbon synapses by regulating the expression of ORC1, which could provide a new idea and targets for the prevention and treatment of ARHL.

## Introduction

Age-related hearing loss (ARHL), also known as presbycusis, is the third most common disability of the elderly in modern society [1]. Although the disorder is multifactorial, ARHL is largely attributed to the irreversible impairment of spiral ganglion neurons (SGNs) [2]. The accumulating data demonstrate that oxidative stress, mitochondrial dysfunction [3], DNA damage accumulation [4, 5], autophagy [6, 7], and inflammation [8] mediate the damage to SGNs, which in turn leads to the progression of hearing loss. However, the exact mechanism involved in SGNs dysfunction of ARHL is still unknown. In addition, recent evidence has shown that normal aging is accompanied by a progressive loss of ribbon synapses, known as synaptopathy, while the destruction of synaptic connections between inner hair cells and cochlear nerve fibers could lead to the loss of cochlear neurons [9]. However, the exact mechanism involved in SGNs dysfunction of ARHL is still unknown.

Fibroblast growth factor 13 (FGF13) is a member of the FGF superfamily. FGF11-14 are intracellular non-secreted proteins known as fibroblast growth factor homologous factors (FHFs) [10]. Although the FHFs unable to activate FGF receptors, as intracellular proteins, they do interact with various intracellular partners. By binding to tubulin, FGF13 is capable of enhancing the stability of microtubules, thereby, regulating the sodium channels of neurons [11] and myocardial cells [12], mediating the migration of cerebral cortex neurons [13], and promoting the regeneration of axon [14]. Besides, FGF13 deficiency caused notable impairment of presynaptic inputs from interneurons of brain [15]. Unfortunately, FGF13 was downregulated in the brain of both Alzheimer’s disease mouse model and patients, while overexpression of FGF13 could significantly improve neuronal damage in a rat model of Alzheimer’s disease [16]. In addition, inner ear FGF13 conditional knockout mice also displayed auditory dysfunction, along with the apoptosis of SGNs [17]. However, how FGF13 varies with age, whether its changes are related to nerve and synaptic damage, and its possible mechanisms are our focus.

In this study, FGF13 was identified downregulated in the SGNs of cochlea with age and negatively correlated with hearing loss. Interestingly, overexpression of FGF13 could statistically alleviate the apoptosis of SGNs and the loss of ribbon synapses, following with changes of ORC1, which suggested that FGF13 plays an important role in elderly hearing loss and is expected to become a new target for prevention and treatment of ARHL.

## Results

### Degeneration of SGNs and ribbon synapses was responsible for hearing loss in ARHL

**Fig. 1:**
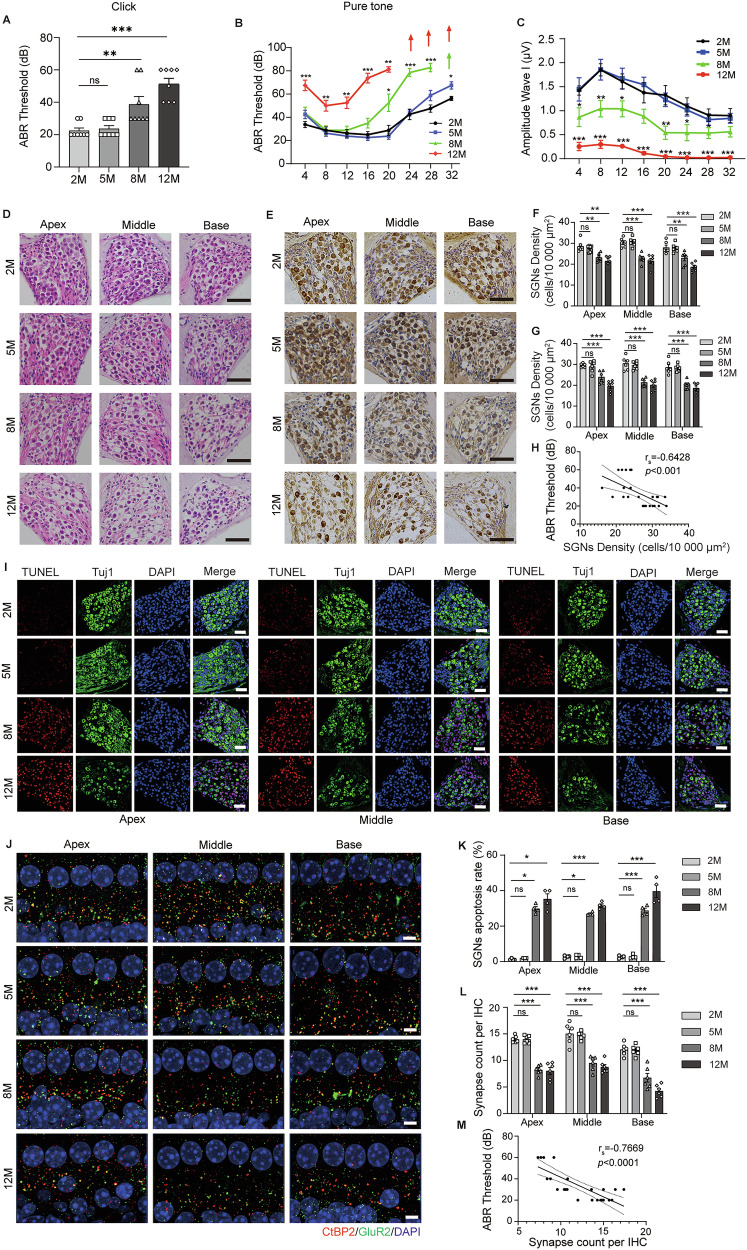
Degeneration of SGNs and ribbon synapses in ARHL mice. **A**, **B** ABR measurements for click and pure tone responses in 2-, 5-, 8-, 12-month-old WT mice (*n* = 8). **C** The ABR peak I amplitudes of 2-, 5-, 8-, 12-month-old mice at 90 dB of all tested frequencies (4–32 kHz). **D** Representative H& E staining of cochlear modiolus in the apical, middle, and basal turns of 2-, 5-, 8-, 12-month-old C57BL/6 J mice. Scale bars: 50 µm. **E** Immunohistochemistry of Tuj1 in the SGNs of 2-, 5-, 8-, 12-month-old C57 mice. Scale bars: 50 µm. **F, G** Quantification of SGNs density depending on the H& E staining and Tuj1 immunolabeling sections (*n* = 6). **I, K** TUNEL staining in the 2-, 5-, 8-, 12-month-old cochlear samples (*n* = 4). Scale bars:38.6 µm. **J, L** Immunofluorescence of IHCs from apex, middle and base of cochlea in 2-, 5-, 8-, 12-month-old C57 mice labeled with anti-CtBP2 (red) and anti-GluR2 (green) (*n* = 6). Scale bars:5 μm. **H, M** Correlation analysis of SGNs density and ribbon synapses with ABR thresholds. Data are represented as mean ± SEM. *p < 0.05, **p < 0.01, ***p < 0.001 versus 2 M group, ns means no significant.

First, we examined the hearing ability in C57BL/6J mice at different age points ranging from 2 to 12 months old. As expected, the hearing thresholds and wave I amplitudes compromised until 8 months (Fig. 1A, C). Moreover, the thresholds exceeded the upper limit of Auditory brainstem response (ABR) at high frequency at 12 months (Fig. 1B), suggesting that auditory function declined upon aging in C57 mice.

**Fig. 2:**
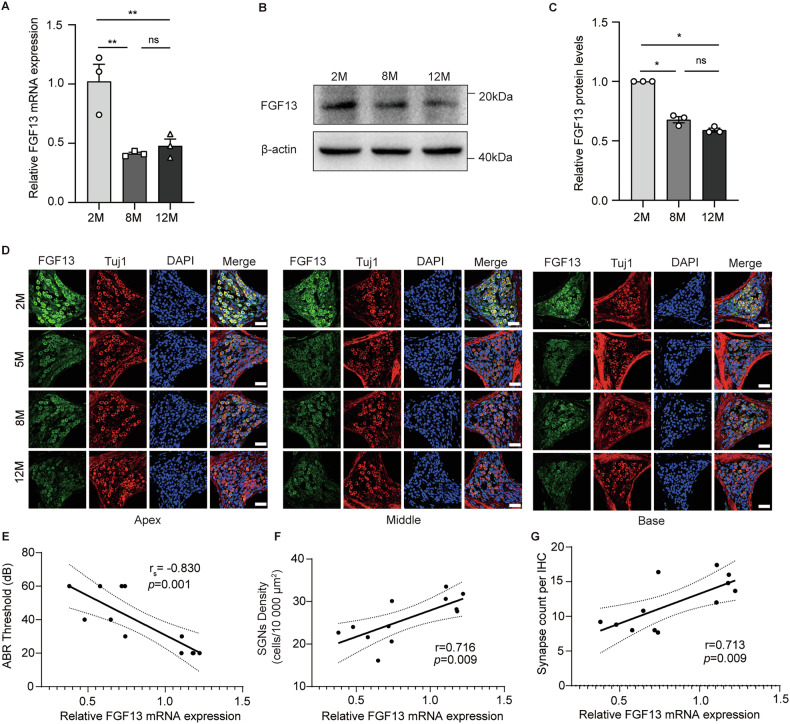
FGF13 expression was decreased in the SGNs of ARHL mice. FGF13 expression was significantly decreased in the cochleae of 8-, and 12-month-old mice at the mRNA and protein levels, as determined by qRT-PCR (**A**) and western blot (**B, C**) (*n* = 3). **D** Representative images of immunofluorescence showing FGF13 expression in SGNs from 2-, 5-, 8-, 12-month-old C57BL/6J mice. Scale bar: 38.6 µm. **E**–**G** Correlation analysis of ABR threshold, SGNs density, and ribbon synapses with the expression of FGF13. Data are represented as mean ± SEM. *p < 0.05, **p < 0.01 versus 2 M group, ns means no significant.

Second, as shown in Fig.1D–G, the density of SGNs was reduced in apex, middle, and base of the cochlea with age and negatively correlated with ABR thresholds (Fig. 2H). Simultaneously, TUNEL results also revealed that the apoptosis rate was increased in SGNs with age (Fig. 1I, K). In addition, hair cell ribbon synapses were labeled with antibodies against CtBP2, a specific presynaptic ribbon protein RIBEYE and postsynaptic GluR2. The number of synapse puncta (CtBP2^+^, GluR2^+^) was significantly reduced in the apical, middle, and basal turns of inner hair cell with age (Fig. 1J, L), and it was negatively associated with ABR thresholds, either. (Fig. 1M). These results indicated that SGNs and ribbon synapses gradually degenerated as the age increasing and they were responsible for auditory dysfunction.

### The expression of FGF13 significantly decreased in the cochlea, especially in SGNs, with age

We further investigated the expression of FGF13 in the cochlea of different ages. As shown in Fig.2A, FGF13 mRNA level was decreased by 60% and 53% in the 8-month-old and 12-month-old mice, respectively. Similarly, western blot results also confirmed that FGF13 protein expression was significantly decreased in the cochlea of aged mice at age-dependent manner (Fig. 2B, C). To further detect the location of FGF13, double immunofluorescence staining was used. As shown in Fig. 2D, FGF13 expression was downregulated in Tuj1-positive cells (Tuj1 was a biomarker of SGNs) with age. Furthermore, the expression of FGF13 was negatively correlated with hearing thresholds (Fig. 2E). At the same time, SGNs density and the number of ribbon synapses were positively correlated with the expression of FGF13 (Fig. 2F, G). Above all, the downregulation of FGF13 was statistically associated with hearing loss and the decrease in the number of SGNs, as well as the ribbon synapses.

### Upregulation of FGF13 could protect against hearing loss in ARHL mice

**Fig. 3:**
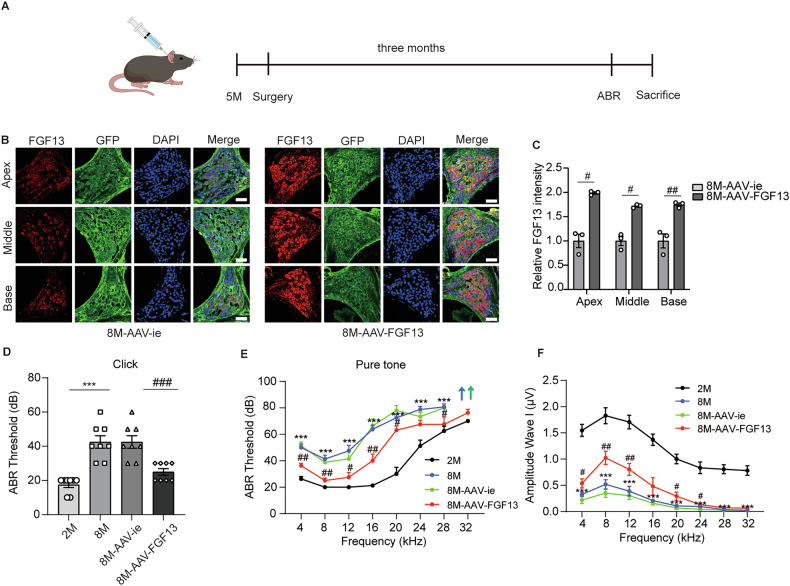
Upregulation of FGF13 significantly alleviated hearing loss. **A** Schematic diagram of animal experiment procedure. **B**, **C** Immunofluorescence for FGF13 in SGNs of 8M-AAV-ie and 8M-AAV-FGF13 groups (*n* = 3). Scale bar: 38.6 µm. **D**, **E** ABR measurements for click and pure tone responses among groups (*n* = 8). **F** The ABR peak I amplitudes were measured among groups at 90 dB of all tested frequencies (4–32 kHz). *p < 0.05, **p < 0.01, ***p < 0.001 versus 2 M group, ^#^*p* < 0.05, ^##^*p* < 0.01, ^###^*p* < 0.001 versus 8M-AAV-ie group. ns means no significant.

**Fig. 4:**
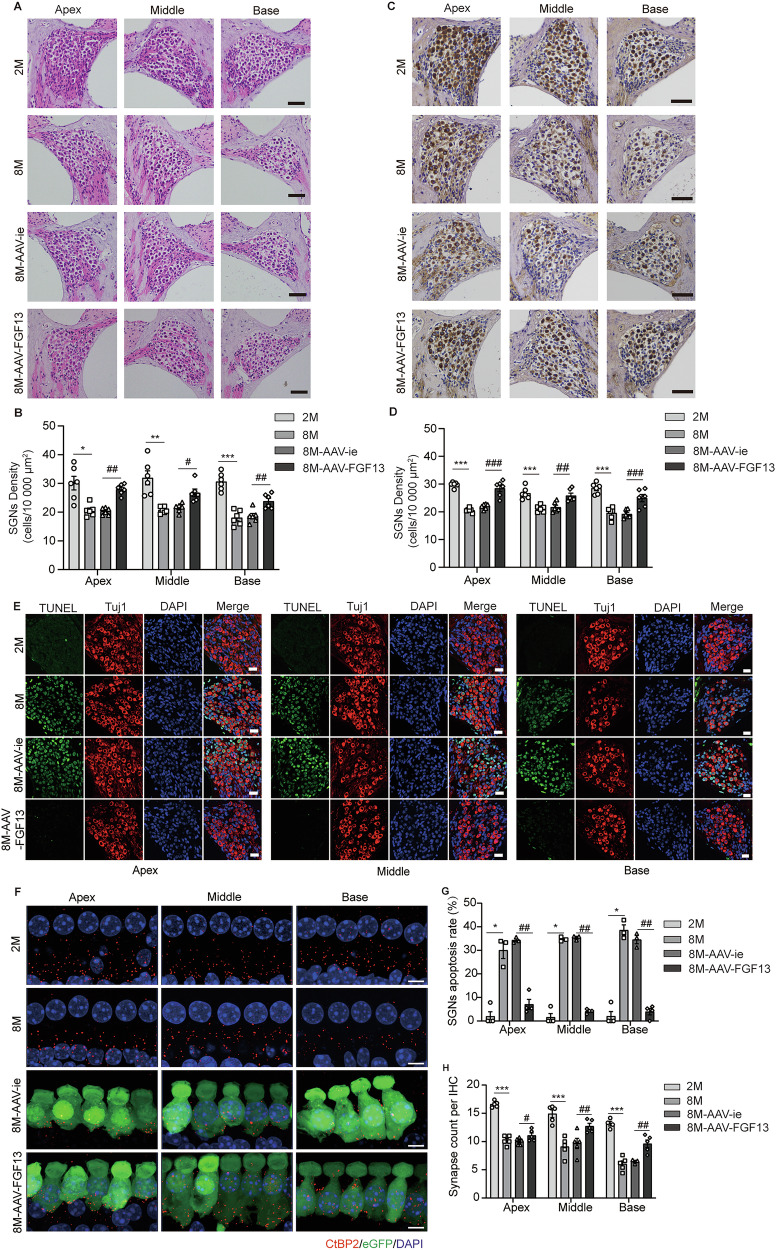
FGF13 upregulation reduced the impairment of SGNs and ribbon synapses. **A** H& E staining of modiolus in the apical, middle, and basal turns among groups. Scale bars: 50 μm. **C** Immunohistochemistry of Tuj1 in the SGNs of different groups. Scale bars: 50 µm. **B**, **D** Quantification of SGNs density depending on the H& E staining and Tuj1 immunolabeling sections (*n* = 6). **E**, **G** TUNEL staining showed the apoptosis of SGNs in apical, middle, and basal turns among groups (n = 3). Scale bars: 38.6 μm. **F**, **H** Immunofluorescence of IHCs from apex, middle and base of cochlea among groups labeled with anti-CtBP2 (red) (n = 5). Scale bars: 5 μm. Data are represented as mean ± SEM. *p < 0.05, **p < 0.01, ***p < 0.001 versus 2 M group, ^#^*p* < 0.05, ^##^*p* < 0.01, ^###^*p* < 0.001 versus 8M-AAV-ie grou*p*.

To further explore the function of FGF13, we delivered FGF13 gene into inner ear through an AAV-ie vector via the posterior semicircular canal (Fig. 3A). As shown in Fig. 3B, C SGNs were efficiently transfected by the AAV-ie vector. Besides, FGF13 expression was significantly upregulated in the AAV-FGF13 group (Fig. 3B, C). Subsequently, ABR was measured in different groups of mice to evaluate the effect of FGF13 on hearing function. The 8-month-old AAV-FGF13 mice exhibited reduced hearing loss compared with age-matched WT and AAV-ie mice (Fig. 3D–F). In agree with this, all three regions of cochlea, i.e., the apex, middle, and base, in the AAV-FGF13 mice, displayed minor loss of SGNs (Fig. 4A–D). Moreover, the rate of apoptosis SGNs also reduced in the AAV-FGF13 mice (Fig. 4C, D). Furthermore, the loss of synapse puncta in IHC was significantly reduced in AAV-FGF13 mice (Fig. 4E, F). Collectively, these data suggested in an opposite direction that downregulation of FGF13 plays an important role in hearing loss in ARHL.

### FGF13 might mediate the loss of SGNs and ribbon synapses by regulating ORC1 in ARHL

**Fig. 5:**
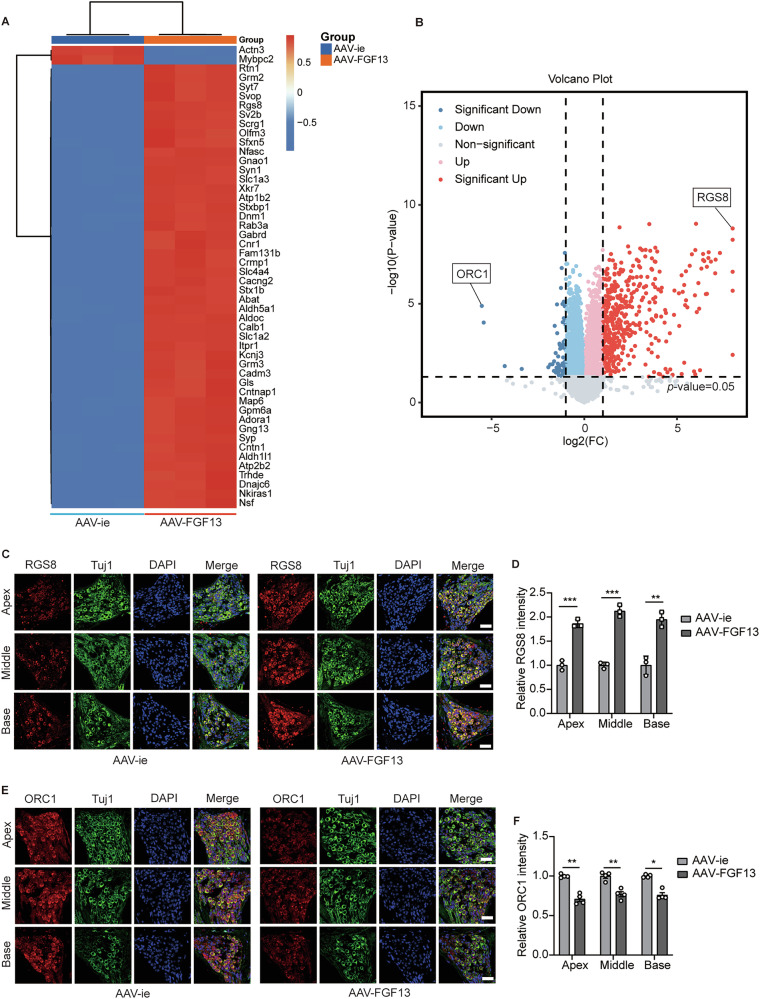
Proteomic analysis revealed FGF13 might mediate the loss of SGNs and ribbon synapses via RGS8 and ORC1. **A** Heat map of differential proteins between AAV-ie and AAV-FGF13 groups. **B** The volcano map of differential proteins expression from DEGs. **C**, **D** Immunofluorescence for RGS8 in SGNs of AAV-ie and AAV-FGF13 groups (*n* = 3). Scale bars: 38.6 μm. **E**, **F** Immunofluorescence for ORC1 in SGNs of AAV-ie and AAV-FGF13 groups (*n* = 4). Scale bars: 38.6 μm. Data are represented as mean ± SEM. *p < 0.05, **p < 0.01, ***p < 0.001 versus AAV-ie group.

**Fig. 6:**
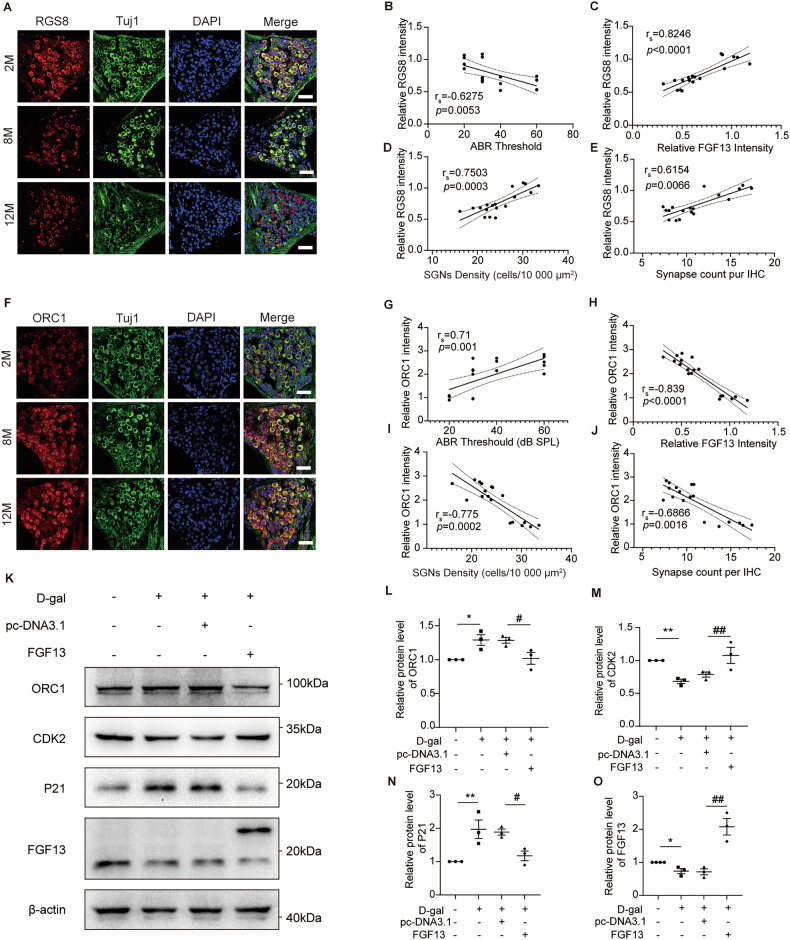
RGS8 and ORC1 is associated with hearing and the impairment of SGNs and ribbon synapses. **A** Immunofluorescence of RGS8 from middle turn of cochlea among 2-, 8-, and 12-month-old mice. Scale bars: 38.6 μm. **B** There was a significantly negative correlation between RGS8 expression and ABR thresholds. **C**–**E** There was a significantly positive correlation among RGS8 expression and FGF13 expression, SGNs density, and ribbon synapses. **F** Immunofluorescence of ORC1 from middle turn of cochlea among 2-, 8-, and 12-month-old mice. Scale bars: 38.6 μm. **G** There was a significantly positive correlation between ORC1 expression and ABR thresholds. **H**–**J** There was a significantly negative correlation among ORC1 expression and FGF13 expression, SGNs density, and ribbon synapses. **K**–**O** Representative western blot and quantitative data showing the expression of ORC1, CDK2, P21 and FGF13 in different groups (n = 3). D-gal means D-galactose. *p < 0.05, **p < 0.01 versus control group, ^#^*p* < 0.05, ^##^*p* < 0.01 versus pcDNA 3.1 + D-gal group. ns means no significant.

Next, in order to elucidate the protective mechanisms of FGF13, we analyzed the differential proteins between the AAV-ie group and the AAV-FGF13 group in mouse cochleae. As illustrated in Fig. 5A, B, 519 differentially expressed genes (DEGs), including 453 upregulated genes and 66 downregulated genes were found between the two groups, in which, RGS8 and ORC1 expression had the most significant changes. The confirmatory analysis by IF staining showed that the expression of RGS8 in the AAV-FGF13 group was increased (Fig. 5C, D), while the expression of ORC1 was significantly reduced in the AAV-FGF13 group (Fig. 6E, F). Then, to further investigate whether RGS8 and ORC1 play a role in age-related hearing loss, we verified the expression of RGS8 and ORC1 in spiral ganglion neurons from different ages of mice. As shown in Fig. 6A, the expression of RGS8 decreased with the age of the mice, while the expression of ORC1 increased with age (Fig. 6F). In addition, correlation analysis showed that the expression of RGS8 was significantly negatively correlated with hearing thresholds (Fig. B), while positively correlated with the expression of FGF13, SGNs density and the number of ribbon synapses (Fig. 6C–E). However, the expression of ORC1 was positively correlated with hearing thresholds (Fig. 6G), while negatively related to the expression of FGF13, SGNs density and the number of ribbon synapses (Fig. 6H–J). Subsequently, we validated these findings in D-galactose induced SH-SY5Y cells. ORC1 expression aligned with in vivo experimental results (Fig. 6K). Besides, ORC1 exhibited the most pronounced downregulation among the identified candidates, prompting further mechanistic investigation. Considering that the expression of ORC1 is closely related to the cell cycle. Besides, apoptosis and cell senescence are also associated with cell cycle arrest. Therefore, we hypothesized that FGF13 may influence the expression of ORC1 by regulating the cell cycle. As seen in Fig. 6K–O, western blot detection found that D-galactose treatment induced increased expression of P21 (a biomarker of cellular senescence), concomitant with decreased levels of CDK2 (a G1/S checkpoint cyclin-dependent kinase). This intervention also resulted in reduced FGF13 expression alongside elevated ORC1 levels. However, when FGF13 was upregulated in SH-SY5Y cells, these effects were reversed, manifesting as attenuated P21 expression, restored CDK2 activity, and diminished ORC1 abundance. Taken together, these results suggested that FGF13 played a role in the development of ARHL partly by regulating ORC1 through modulating the cell cycle.

## Discussion

In the present study, we found for the first time that FGF13 expression was significantly decreased in the SGNs of cochlea in ARHL mice and the downregulation of FGF13 was related to hearing loss and the degeneration of SGNs and ribbon synapses. Recent studies also showed that patients with Wildervanck syndrome [18] and congenital hypertrichosis [19] carrying FGF13 functional deficiencies universally manifested deafness phenotype. Besides, mice with inner ear-specific knockout of FGF13 exhibited significant hearing loss at three months of age, accompanied by reduced amplitude and prolonged latency of ABR wave I, concomitant with apoptotic degeneration of SGNs [17]. Collectively, these data suggested that FGF13 demonstrated critical functional significance in auditory physiology.

In previous studies, the apoptosis of SGNs [3] and the loss of ribbon synapses [20] were demonstrated in ARHL. Due to the non-regenerative nature of SGNs, the apoptosis of these neural cells leads to irreversible sensorineural hearing loss, which substantially compromises the therapeutic efficacy of hearing aids and cochlear implants [21]. In addition, degeneration of synaptic connection affects the normal encoding of the temporal properties of sound, leading to decreased speech perception among the elderly population in noisy environment [22]. In this study, we demonstrated that upregulation of FGF13 in cochlear SGNs significantly improved auditory function in 8-month-old ARHL mice. This protective effect may be mediated through dual mechanisms. Firstly, FGF13 overexpression reduced the apoptosis rate of SGNs, suggesting that its neuroprotective role via anti-apoptosis pathways. Consistent with our study, Yu et al demonstrated that FGF13 knockout in the inner ear upregulated pro-apoptotic genes, such as BAX, Caspase 3, CYCS. While, BCL2, a kind of anti-apoptotic gene, was downregulated. Additionally, FGF13-deficient mice exhibited elevated TUNEL-positive rates in SGNs [17]. BUBLIK et al. further reported that FGF13 knockdown in cancer cells induced apoptosis through ribosomal RNA transcriptional suppression and proteostasis stress, culminating in reactive oxygen species (ROS) accumulation [23]. However, tissue-specific effects were observed, as FGF13 ablation in glomerular endothelial cells ameliorated type 2 diabetic nephropathy by inhibiting apoptosis [24]. Secondly, FGF13 could preserve cochlear ribbon synapses. In this study, FGF13 upregulation restored ribbon synapses density across apical, middle, and basal cochlear turns, aligning with emerging evidence of FGF family members enhancing synaptic plasticity. Ram et al found that FGF13 deficiency reduced inhibitory synaptic inputs in hippocampal neurons, impairing interneuron excitability and precipitating epileptogenesis [25]. Similarly, conditional FGF13 knockout in cortical chandelier cells decreased presynaptic input density [26]. Parallel findings in the FGF family include FGF22-mediated protection of ribbon synapses against ototoxic damage. FGF22−/− mice exhibited diminished ABR wave I amplitudes, synaptic vesicle depletion, and impaired exocytosis efficiency [27]. Taken together, these findings suggested that FGF13 played a role in cell apoptosis and synapse transmission. However, our study only focused on synaptic quantity, future investigations should employ patch-clamp techniques to characterize FGF13’s effects on ribbon synapse electrophysiology.

Due to the absence of a secretory signal sequence, FGF13 is not secreted extracellularly like other FGFs to bind and activate FGF receptors [28]. Instead, it exerts its unique functions through intracellular interactions with multiple proteins. The most well-characterized role of FGF13 is its ability to bind tubulin, promoting tubulin polymerization and stabilizing microtubules, thereby mediating diverse physiological effects across tissues [12]. In dorsal root ganglion (DRG) neurons, FGF13 stabilizes microtubules to selectively enhance TRPV1 channel activity. Genetic ablation of FGF13 in DRG neurons reduces TRPV1 activation, attenuates calcium signaling and neuronal excitability, and alleviates itch sensation [10]. Furthermore, FGF13 contributes to cardiac fibrosis via microtubule-dependent mechanisms. Knockout of FGF13 in cardiac fibroblasts suppresses fibroblast activation and function by impairing microtubule stability and inhibiting ROCK signaling pathway activation, ultimately ameliorating cardiac fibrosis [29]. Recent studies have identified an interaction between FGF13 and Parkin. In glomerular endothelial cells of type 2 diabetic nephropathy (T2DN), FGF13 expression is upregulated. Depletion of FGF13 modulates mitochondrial homeostasis during T2DN progression by leveraging Parkin’s dual roles in promoting mitophagy and suppressing apoptosis, thereby mitigating renal injury [24]. Given the multifaceted intracellular protein interactions of FGF13, in this study, we performed proteomic sequencing on cochlear tissues from FGF13 overexpressing mice and empty vector controls to further elucidate the mechanisms underlying its protective functions. Our data supported that ORC1 and RGS8 may be implicated in the process. Notably, ORC1 exhibited the most pronounced downregulation among the identified candidates, prompting further mechanistic investigation. ORC1 is the biggest subunit of origin recognition complex (ORC) [30]. Eukaryotic DNA replication begins with the binding of ORC to DNA [31]. In human cells, the level of ORC1 oscillates, a phenomenon termed “the ORC1 cycle”. ORC1 starts to accumulate in mid-G1 phase, reaches a peak at the G1/S boundary. At the onset of S phase, ORC1 is ubiquitinated and degraded by the SCF^SKP2^-activated proteasome complex. Then, in late G2 phase, ORC1 is resynthesized, binding to chromosome as cells progress into mitosis [32]. In this study, ORC1 expression was significantly upregulated in the cochlear SGNs of aged mice and positively correlated with elevated auditory thresholds, degeneration of SGNs, and ribbon synapses loss. Similar to our finding, recent studies demonstrated that overexpression of ORC1 induced apoptosis in HeLa cells [33]. In addition, elevated expression of the DNA replication licensing protein ORC1 exacerbated DNA damage response, upregulated P21 expression, and triggered cellular senescence [34]. Overly, these findings suggest that ORC1 may contribute to the pathological progression of ARHL by promoting neurodegenerative mechanisms.

ORC1 protein levels are tightly regulated in a cell cycle-dependent manner [35]. While cellular senescence is a hallmark of aging that is characterized by terminal cell cycle arrest [36]. P21, a well-established senescence-associated marker, negatively regulates cyclin-dependent kinases (CDKs) to induce cell cycle arrest [37]. In this study, we confirmed that FGF13 upregulation suppressed expression of P21 while elevating levels of CDK2, a critical G1/S checkpoint regulator. These findings collectively indicate that FGF13 overexpression alleviates cell cycle arrest. Align with our study, in non-small cell lung cancer, high levels of FGF13 inhibited the activity of P21 and P27, thus, enhancing the process of transition from G1 to S phase and promoting A549 cells proliferation [38]. Besides, in human cutaneous melanoma cells, upregulation of FGF13 leads to fewer cells arrest at the G0/G1 phase and more cells arrest at S phase, as well as a decline in the apoptosis of cells [39]. Taken together, these findings suggested FGF13 modulated ORC1 expression through cell cycle regulatory mechanisms.

In conclusion, our findings demonstrated that FGF13 is of great importance in maintaining the function of SGNs and ribbon synapses in aged mice. The deficiency of FGF13 gives rise to the apoptosis of SGNs and the impairment of ribbon synapses, which in turn further aggravates hearing. Our study contributes to the understanding of pathogenesis of presbycusis and might provide novel insight for the treatment of ARHL.

## Materials and methods

### Animals

All experimental animal protocols were performed following the Animal Care and Ethical Committee of The Second Hospital of Hebei Medical University (Approval Letter No. 2022-AE308). 2-, 5-, 8-, 12-month-old male C57BL/6J mice used in this study were purchased from Beijing HFK Biotechnology and kept in a quiet environment with enough water and food under a standard 12 h: 12 h light-dark cycle. In addition, male C57BL/6J mice, aged 5 months, were divided into three groups: normal control group (*n* = 8), AAV-ie group (*n* = 12), and AAV-FGF13 group (*n* = 12). Two-month-old mice serve as young control group (*n* = 8). The mice in the AAV-ie group, and AAV-FGF13 group were injected with 2 μl 1 × 10^13^ vg/mL of adenoviruses (PackGene Biotech, Guangzhou, China) in the left ear. The AAV-ie vector carrying FGF13, or empty vector with an enhanced green fluorescent protein. After 3 months, the mice were sacrificed at the age of 8-month-old.

### ABR testing

ABR measurements were performed as previous described [40]. Briefly, mice were anesthetized with ketamine (100 mg/kg) and xylazine (10 mg/kg). Three silver wire electrodes were inserted subcutaneously at the vertex of the skull (reference), the mastoid process of the tested ear (recording) and the mastoid process of contralateral ear (ground). ABR was measured using a TDT system (Tucker-Davis Technologies, Gainesville, FL, USA). Responses were measured for clicks and pure tones at various frequencies. The hearing threshold was determined as the lowest stimulus level at which a repeatable Wave II could be visually identified. Amplitudes of Wave I at 90 dB were evaluated.

### SGNs morphometry and counting

The cochleae were fixed in 4% paraformaldehyde and decalcified in 10% EDTA at 4 °C. The decalcified cochleae were dehydrated by gradient alcohol and then embedded in paraffin, and cut into 4 μm sections. To evaluate SGNs morphometry and density, cochlear sections were stained with immunohistochemistry as well as hematoxylin and eosin. Immunohistochemistry staining was performed as previous described. Briefly, antigen recovery was performed using a pressure cooker, the primary antibodies concentration was anti-Tuj1(1:200, Biolegend, #801201, California, USA). Images were captured using an Olympus microscope (OLYMPUS, BX71, Tokyo, Japan). Six cochlear samples from each subgroup were used for SGNs counting. The Rosenthal’s canal was divided into three regions: the apex, middle, and base. The number of SGNs in each section was divided by the area of Rosenthal’s canal, and the total number of SGNs was counted using Image J software. Finally, the density of SGNs was calculated within the unit area (10,000 μm^2^) from each section of cochlea.

### Immunofluorescence

Cochlear frozen sections and whole mount preparations were stained following a previously established protocol [40]. The antibody concentration was anti-CtBP2 (1:250, Invitrogen, #PA5-79086, Waltham, Massachusetts, USA), anti-GluR2 (1:200, Invitrogen, #32-0300), anti-FGF13 (1:200, Gene Tex, #GTX101008, Irvine, California, USA), anti-Tuj1 (1:200, Biolegend, #801201), anti-GFP (1:200, Proteintech, #66002-1-lg, Chicago, USA), anti-RGS8 (1:50, Proteintech, #27294-1-AP), and anti-ORC1 (1:50, Absin, #abs146248, Shanghai, China). Images were obtained with a confocal laser-scanning microscope (Zeiss 900 confocal system).

### TUNEL staining

TUNEL staining was conducted on paraffin sections utilizing the Kit (Vazyme, #A112, Nanjing, China) following the manufacturer’s protocol. Sections were then mounted with an anti-fluorescence quenching agent containing DAPI. The percentages of TUNEL positive cells were quantified by Image J software.

### Quantitative real-time PCR

The total RNA sample from mouse cochlea (5 mice were used for each sample) was extracted at 4°C using Trizol RNA isolating reagent (Thermo Fisher Scientific, Waltham, MA, USA) according to the established procedures. As in the reverse-transcription kit manual (Roche, Mannheim, Germany), equal amounts of RNA (1000 ng) were synthesized to cDNA using the random hexamers and superscript II reverse transcriptase. Quantitative amplification was performed in triplicate on a Bio-Rad CFX Connect Real-Time PCR System. The relative gene expression was analyzed by calculating the respective GAPDH using the formula: 2^-△△ct.^ Primer sequences were as follows: FGF13 forward primer 5′-CAGCCGACAAGGCTACCAC-3’ and reverse primer 5′-GTTCCGAGGTGTACAAGTATCC-3′. GAPDH forward primer 5′-TGTCAGCAATGCATCCTGCA-3’ and reverse primer 5′-CCGTTCAGCTGGGATGAC.

### Western blot

The proteins from mouse cochlea tissues (2 mice were used for each sample) or cells were extracted with strong RIPA lysis buffer containing PMSF (Solarbio, #R0010, Beijing, China). Then the protein concentration of each sample was measured by BCA kit following manufacturer’s instruction. Equal amounts of protein (30 μg) were separated using 10% or 12% SDS-PAGE gels depending on the molecular weight and then blotted onto PVDF membranes. After being blocked, the membranes were incubated overnight at 4 °C with a 1:1000 dilution of FGF13 (Gene Tex, #GTX101008), 1:1000 dilution of ORC1 (ABclonal, #A14756, Wuhan, China), 1:1000 dilution of P21 (Proteintech, #10355-1-AP), 1:1000 dilution of CDK2 (Cell Signaling Technology, #2546, Danvers, MA, USA), and 1:4000 dilution of β-actin (Proteintech, #20536-1-AP). Next day, the membranes were incubated with the secondary antibodies conjugated with horseradish peroxidase. At last, the visualization and data processing were performed by a ChemiDoc XRS system (Bio-Rad). All experiments were repeated at least three times.

### Proteomics sequencing

Cochleae were divided into the AAV-ie group and the AAV-FGF13 group (6 animals per group). The proteomic sequencing was performed by Shanghai OE Biotech Co., Ltd. (Shanghai, China) using liquid chromatography-tandem mass spectrometry (LC-MS/MS) following standard procedures. In brief, total cochlear proteins were extracted, followed by concentration determination, SDS-polyacrylamide gel electrophoresis, trypsin digestion, and peptide desalting. All data were acquired using a Thermo Scientific Vanquish Neo UHPLC system coupled to an Orbitrap Astral mass spectrometer with a Thermo Scientific Easy-spray source.

### Cell line and groups

The human neuroblastoma cell line (SH-SY5Y cells, purchased from the Ethephon Biotechnology, Shanghai, China) was maintained in Dulbecco’s modified Eagle’s medium (Gibco, Shanghai, China) with 10% fetal bovine serum (Lonsera, #S711-001S, Uruguay), supplemented with 1% Penicillin-Streptomycin (Solarbio, #P1400) at 37°C in 5% CO_2_. To elucidate the function of FGF13, SH-SY5Y cells were divided into four groups: control group, D-galactose group, pcDNA 3.1 + D-galactose group and pcDNA 3.1-FGF13 + D-galactose group.

### Cell transfection and drug treatment

The FGF13 overexpression plasmid (NM_033642) was purchased from Youbio (Changsha, China). According to the manufacturer’s instructions, cells were transfected using Lipomaster 2000 Transfection Reagent (Vazyme, #TL201). After transfection for 24 h, the medium was replaced with 300 mM D-galactose (Sigma, #G5388, USA) and incubated for an additional 24 h.

### Statistical Analysis

The data were presented as the mean ± SEM. SPSS 21.0 (SPSS, Inc., Chicago) was used for data analysis. Student’s *t*-test and Mann-Whitney nonparametric tests were performed for comparisons between the two groups. For comparisons more than two groups, statistical analysis was performed using one-way analysis of variance (ANOVA) followed by Bonferroni post hoc test. Spearman’s test was performed to assess the correlation between two sets of data. A *p* value < 0.05 was considered statistically significant.

## Supplementary information

Original western blots

## Data Availability

The data supporting the results of this study are available on reasonable request from the corresponding author.
