# Supplementary material for: FGF13 prevents age-related hearing loss by protecting spiral ganglion neurons and ribbon synapses from injury
Source: Cell Death Discov. 2025 Jul 5;11:307. doi: 10.1038/s41420-025-02607-5 (PMC12228769; doi:10.1038/s41420-025-02607-5)

**Supplementary Material: Uncropped immunoblots of Fig. 2B**

Anti-FGF13


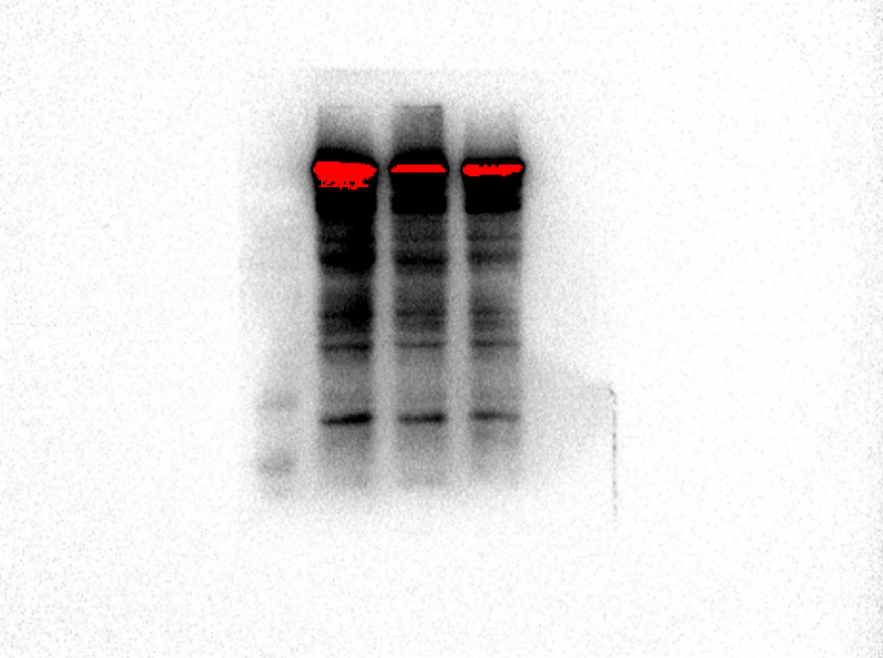


FGF13

17kDa


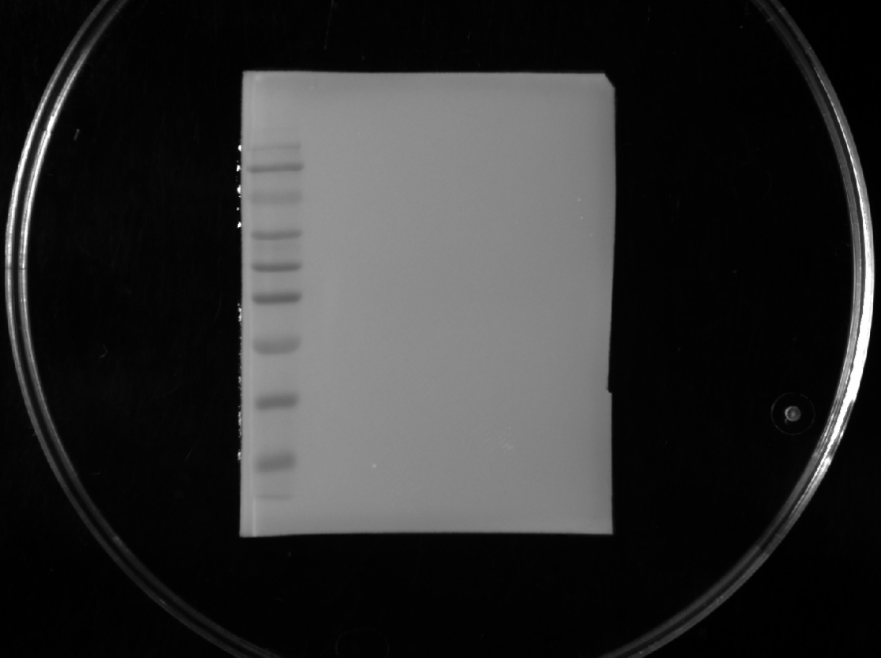


Anti-β-actin


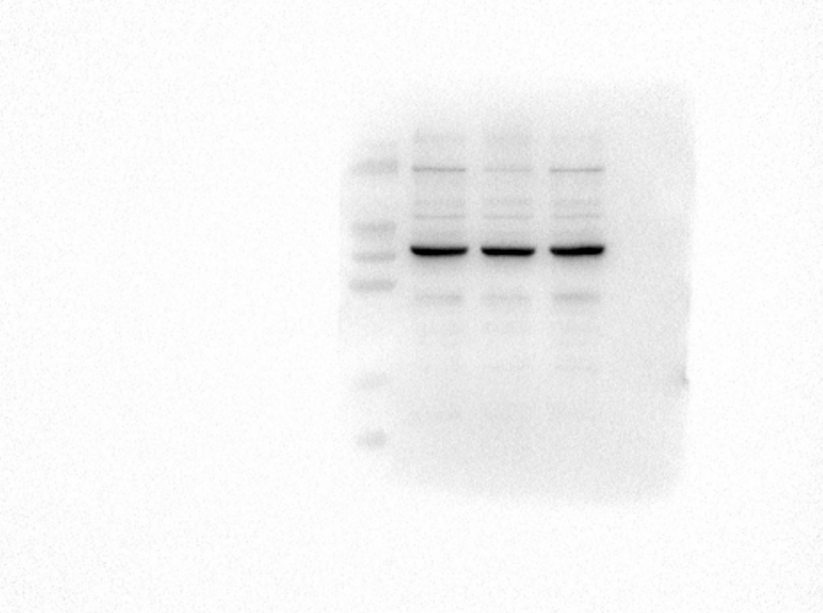


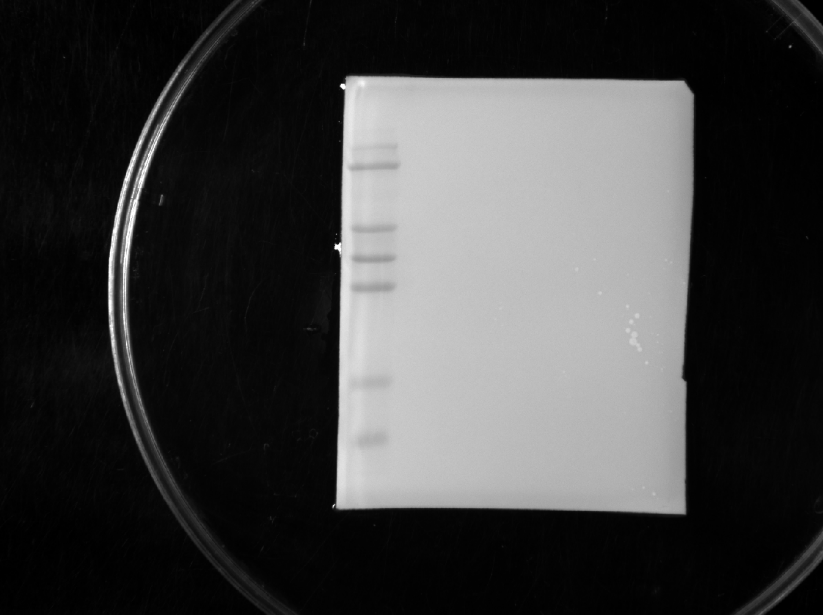


**Uncropped immunoblots of Fig. 4K**

Anti-ORC1


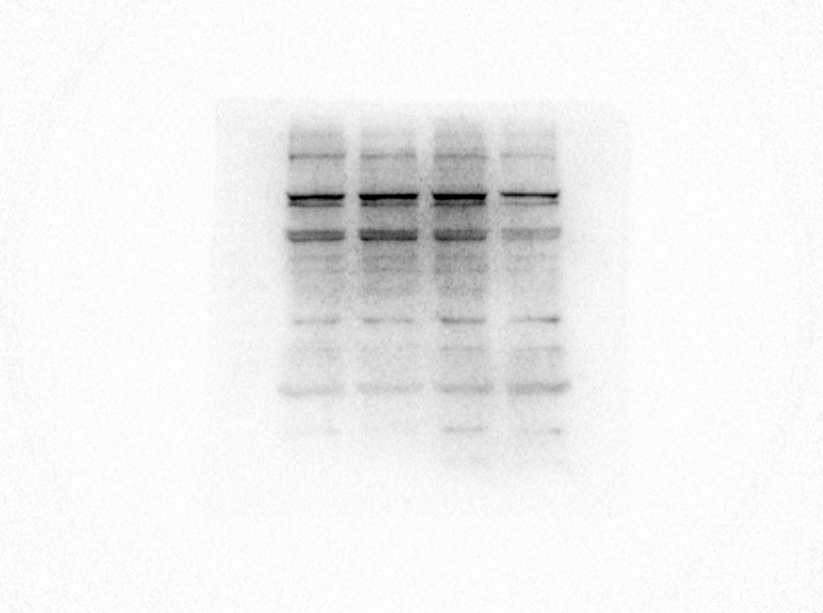


ORC1

97kDa


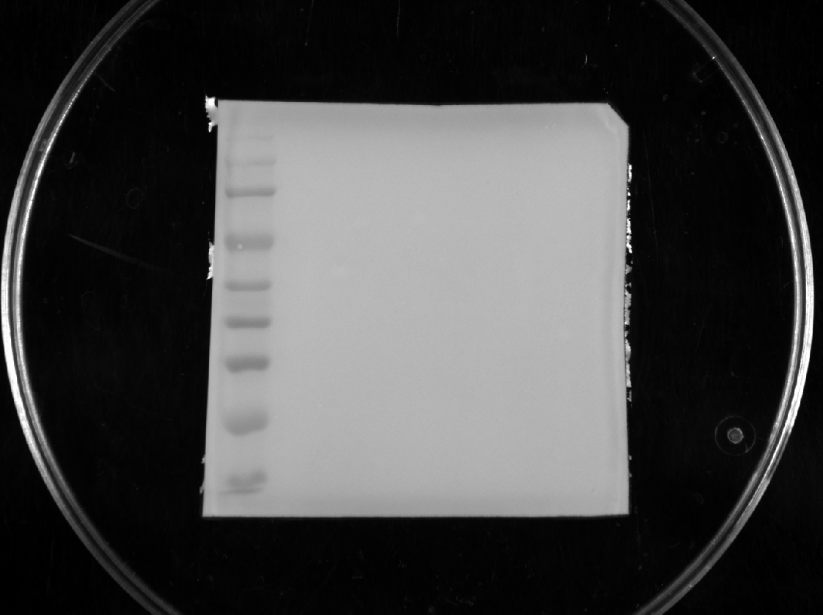


Anti-β-actin


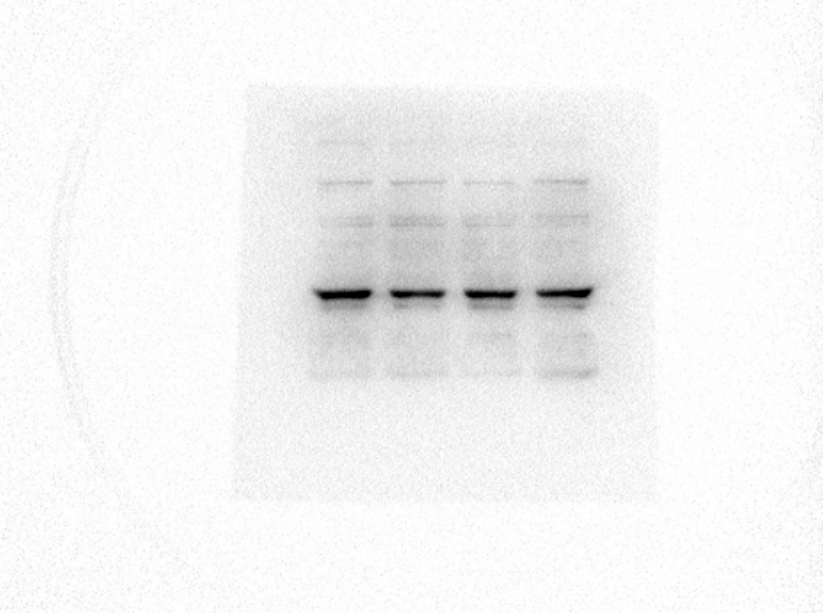


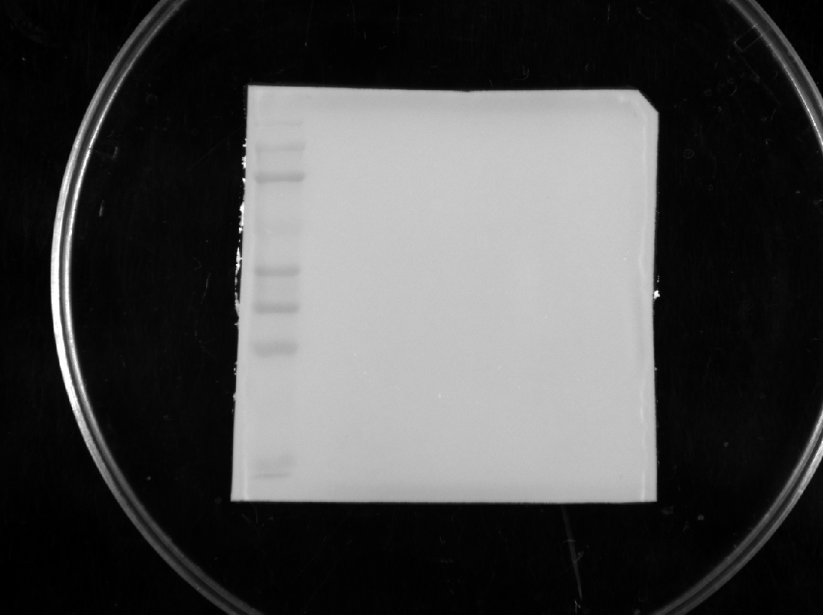


Anti-CDK2


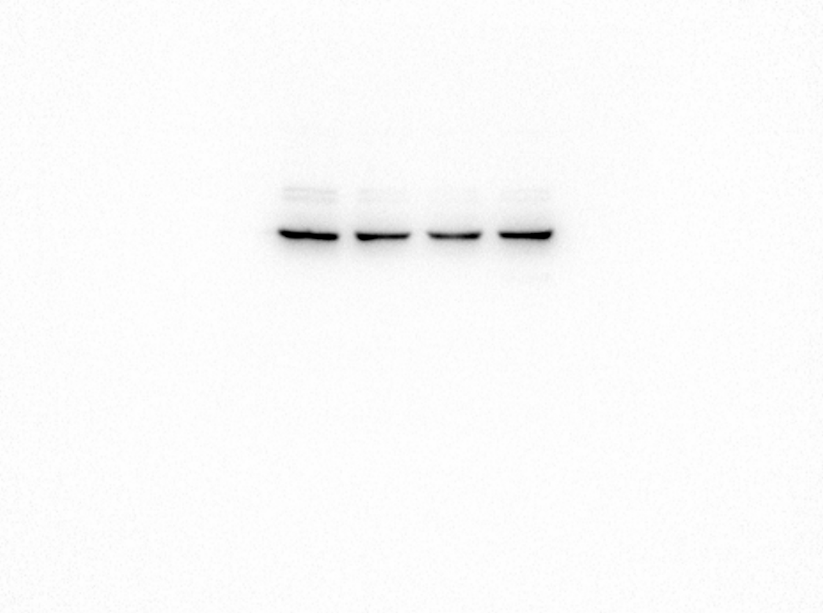


33kDa

CDK2


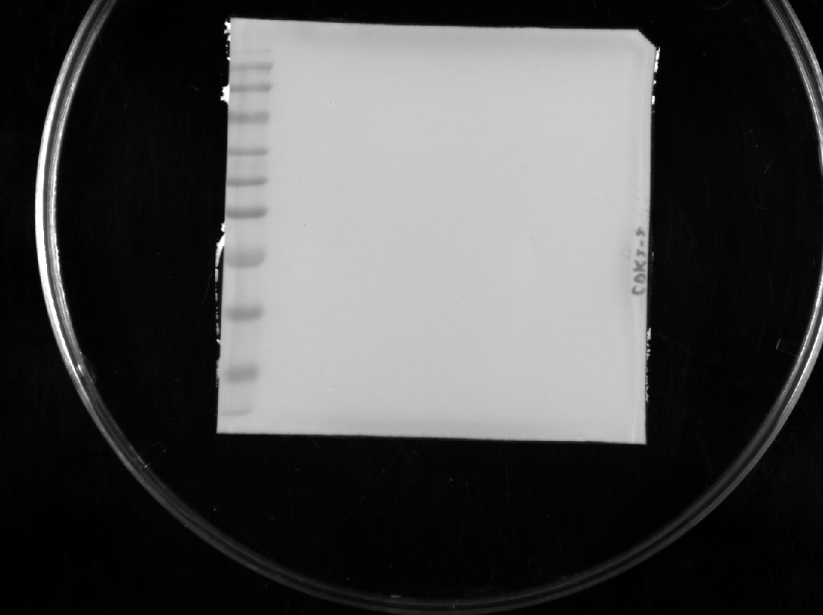


Anti-β-actin


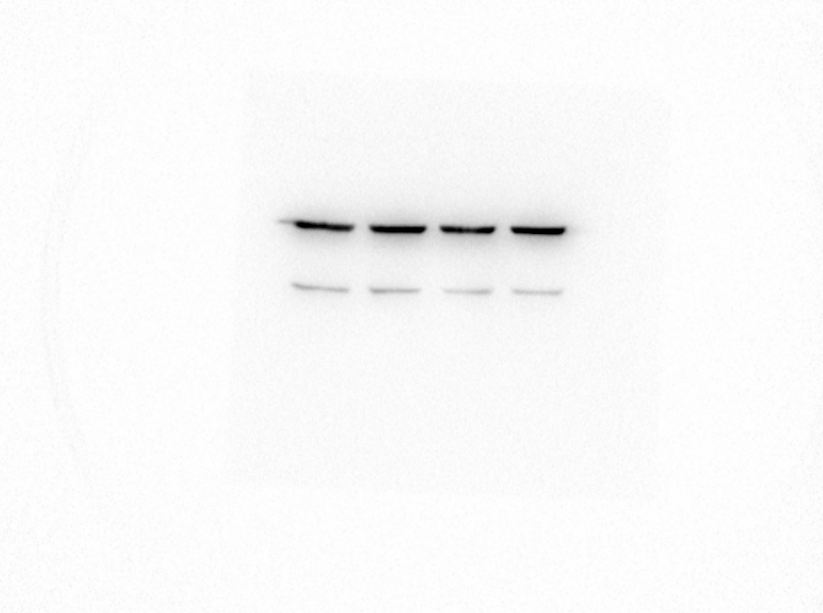


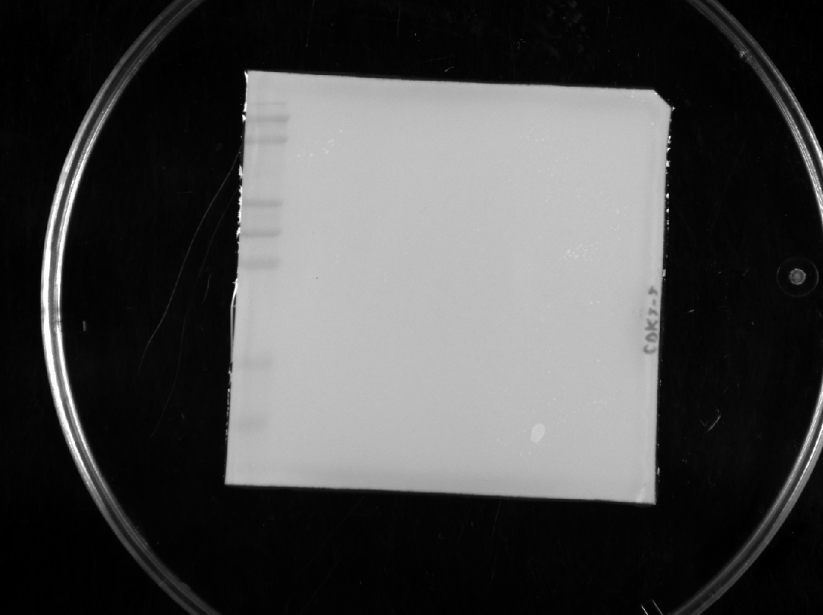


Anti-P21


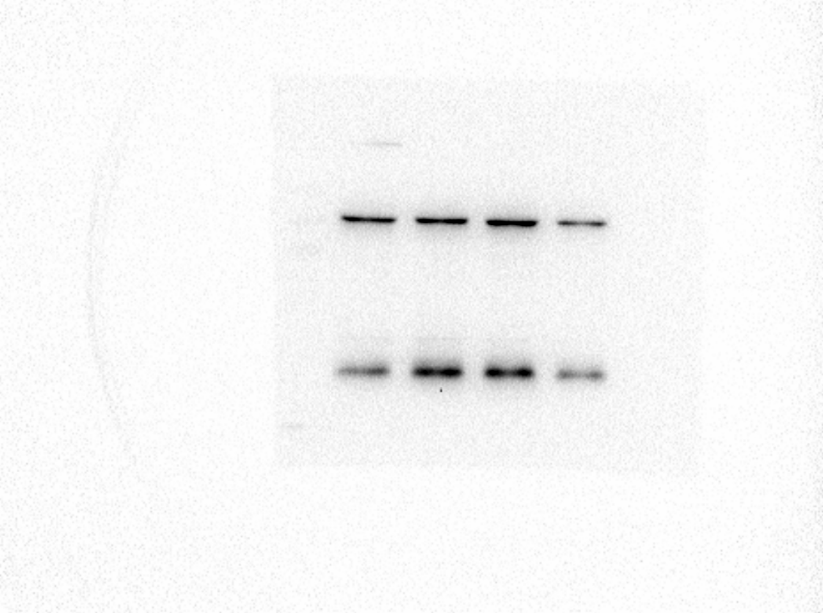


18kDa

P21


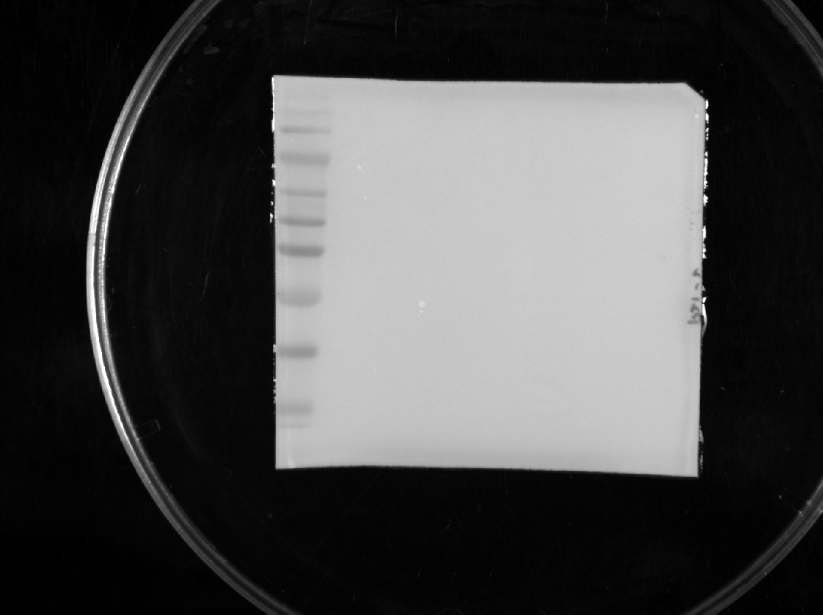


Anti-β-actin


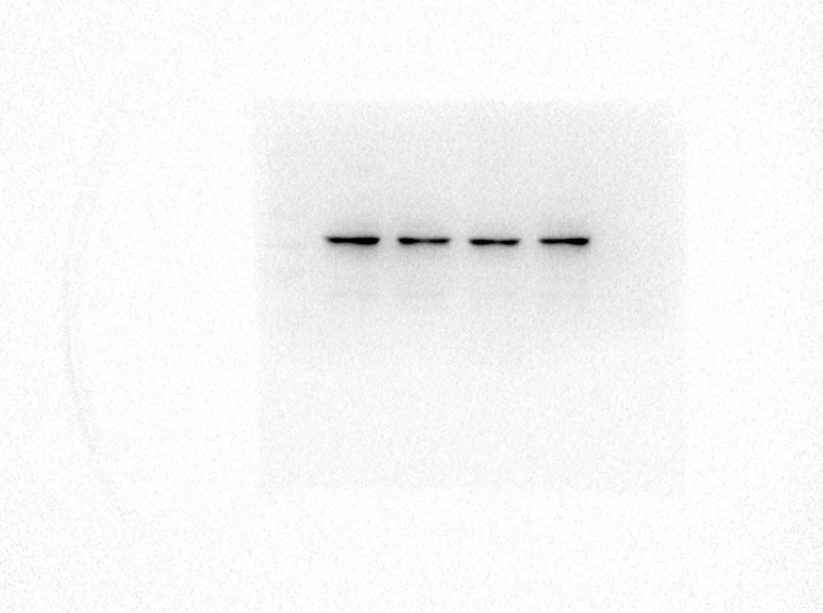

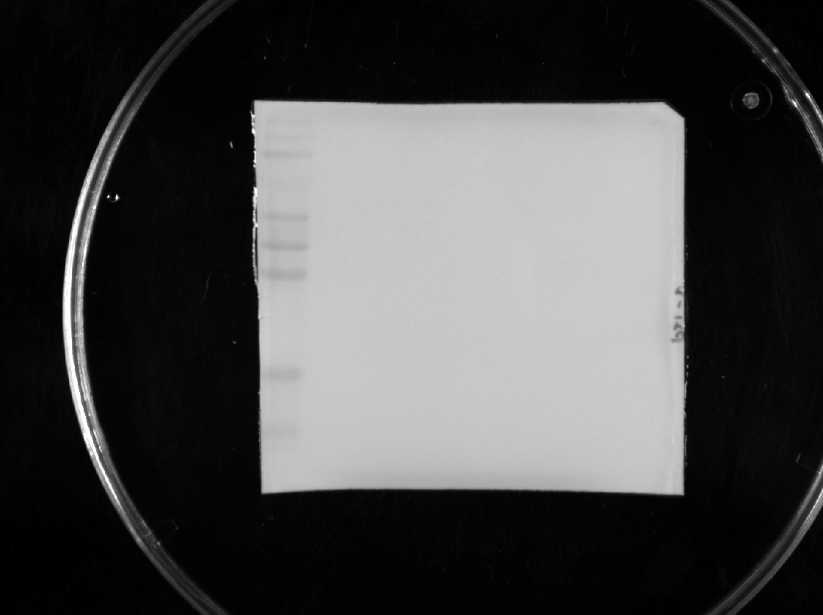


Anti-FGF13


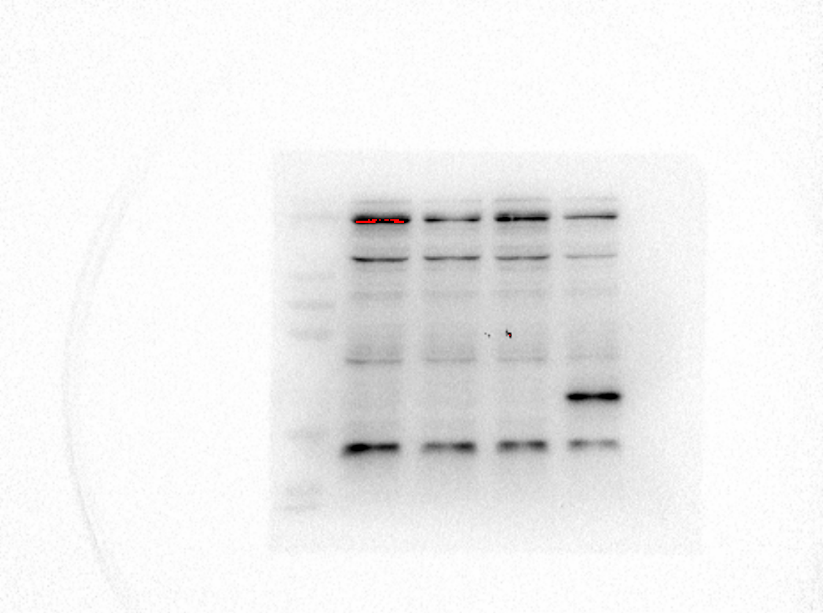


17kDa

FGF13


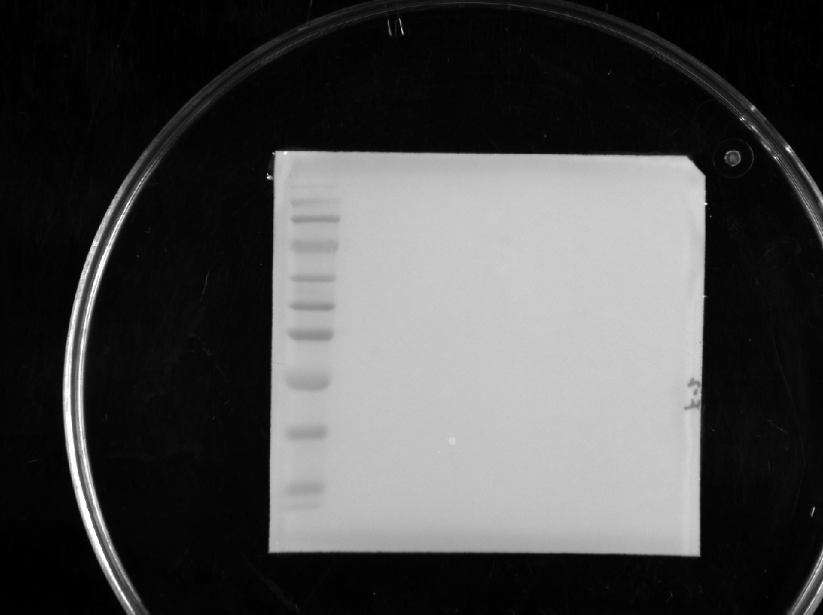


Anti-β-actin


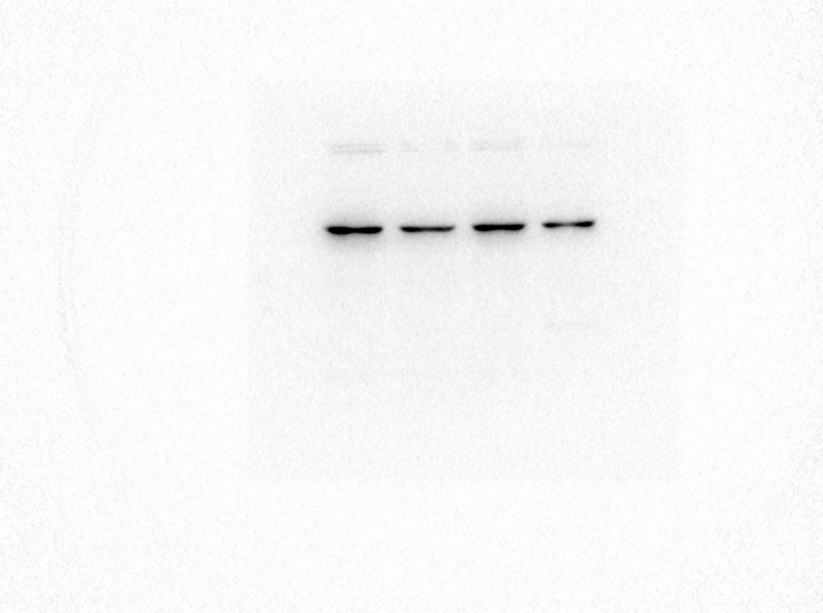


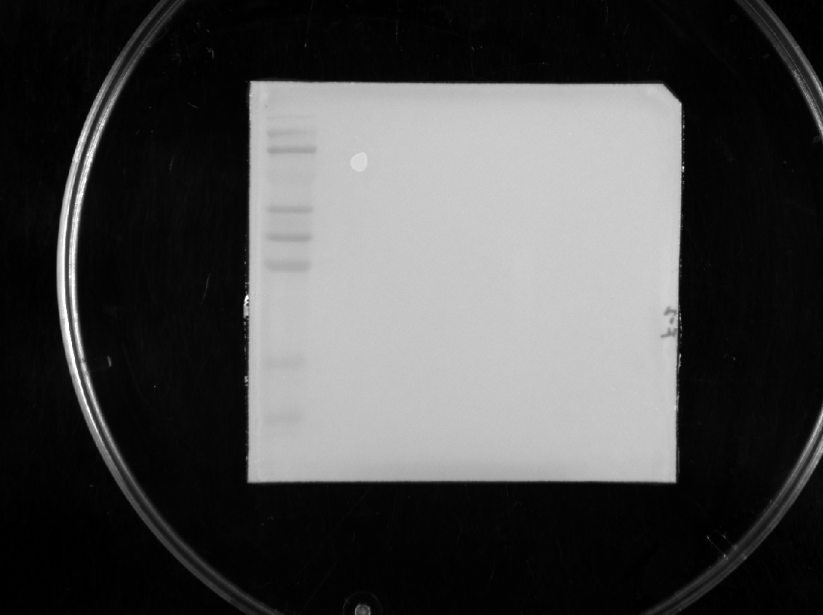

Supplement: Supplementary file 1 — Original western blots [file 41420_2025_2607_MOESM1_ESM.docx]
